# Nrf2 modulates cytosolic and mitochondrial calcium signal

**DOI:** 10.1016/j.redox.2026.104213

**Published:** 2026-05-15

**Authors:** Alessandra Preziuso, Artyom Y. Baev, Fozila R. Rustamova, Sharadha Dayalan Naidu, Lauren Millichap, Plamena R. Angelova, Vincenzo Lariccia, Albena T. Dinkova-Kostova, Andrey Y. Abramov

**Affiliations:** a Department of Biomedical Sciences and Public Health, School of Medicine, University "Politecnica delle Marche", Via Tronto 10/A, Ancona, 60126, Italy; b Centre for Advanced Technologies, Academy of Science of the Republic of Uzbekistan, Tashkent, Uzbekistan; c Jacqui Wood Cancer Centre, Division of Cancer Research, Faculty of Health, University of Dundee, Dundee, UK; d Department of Clinical and Movement Neurosciences, UCL Queen Square Institute of Neurology, Queen Square, London, WC1N 3BG, UK

**Keywords:** Nrf2, Keap1, Calcium signal, Mitochondria, Neuron, Astrocyte

## Abstract

Nrf2 is a transcription factor which regulates ∼1% of the mammalian genome and is responsible for orchestrating the cellular defense against oxidative, inflammatory and metabolic stress. Calcium (Ca^2+^) is a ubiquitous intracellular messenger which controls most cellular processes, from fertilization to cell death. Nrf2 and Ca^2+^ are involved in a large number of similar physiological processes, but it is not clear if they can regulate each other. Here, using primary co-cultures of neurons and astrocytes we asked if Nrf2 activation or deficiency alters physiological Ca^2+^ signaling and mitochondrial Ca^2+^ handling in brain cells. We found that activation of Nrf2 leads to an increase in the amplitude of Ca^2+^ peak and a faster Ca^2+^efflux in response to glutamate and ATP in neurons and astrocytes. Interestingly, Nrf2-deficient neurons and astrocytes also had higher Ca^2+^ peaks in response to glutamate and ATP, but the recovery in neurons was significantly delayed. Genetic (Keap1-knockdown) or pharmacological (ovameloxolone, RTA-408) activation of Nrf2 increases mitochondrial Ca^2+^ uptake and mitochondrial Ca^2+^ capacity, and this correlates with increased activity of the Na^+^/Ca^2+^/Li^+^ exchanger (NCLX) and inhibition of the mitochondrial permeability transition pore (mPTP). Conversely, mitochondria in neurons and astrocytes from Nrf2-knockout mice had a lower Ca^2+^ uptake, lower mitochondrial Ca^2+^ capacity and lower mitochondrial Ca^2+^efflux, making these cell vulnerable to Ca^2+^-induced cell death. Thus, Nrf2 modulates cytosolic calcium signaling and activates the mitochondrial NCLX, increasing the mitochondrial Ca^2+^ capacity, which adds another critical aspect to the multifaceted nature of Nrf2-mediated cytoprotection.

## 1 Introduction

Ubiquitous intracellular messenger Ca^2+^ is not only the regulator of all physiological processes in mammalian cells but also a regulator of the other signaling cascades (such as phospholipases activity, energy metabolism), and any disturbances can lead to Ca^2+^ disbalance, and consequently, various pathologies and cell death [1,2].

In brain cells, such as neurons and glia, activation of Ca^2+^ signaling is induced by specific receptors which produce ionotropic (plasmalemmal Ca^2+^ channel activation) or metabotropic - metabolic cascade activation resulting in release of Ca^2+^ from the endoplasmic reticulum (ER) to the cytoplasm [3]. The extrusion of Ca^2+^ out of the cytoplasm is an energy-dependent process that requires plasmalemmal Ca^2+^-ATPase or Na^+^/Ca^2+^ exchanger, and calcium refiling of ER is operated by sarco-*endo*-plasmic reticulum Ca^2+^-ATPase (SERCA) [4,5]. Mitochondria play a dual role in regulating intracellular calcium signaling. Mitochondrial Ca^2+^ uptake stimulates several Ca^2+^-sensitive matrix enzymes, enhancing oxidative metabolism and ATP production required both for the restoration of Ca^2+^ homeostasis and for Ca^2+^-dependent cellular processes. In addition, transient mitochondrial Ca^2+^ buffering reduces the cytosolic Ca^2+^ load and, under conditions of rapid and large-amplitude Ca^2+^ signals, extends the time available for normalization of intracellular Ca^2+^ levels [6]. Mitochondria uptake Ca^2+^ via electrogenic mitochondrial calcium uniporter and remove it using mitochondrial Na^+^/Ca^2+^ exchanger [7,8]. Mitochondrial Ca^2+^ efflux in brain cells is Na ^+^ dependent process in exchanger identified as NCLX, recently was shown that TMEM65 can act as Na^+^/Ca^2+^ exchanger or as a component NCLX [9,10]. In non-excitable cells mitochondrial calcium efflux can be Na^+^-independent and act through Ca^2+^/H^+^ exchanger [11]. Dysfunction of mitochondrial calcium homeostasis leads to mitochondrial Ca^2+^ overload, and in combination with increase production of reactive oxygen species (ROS), to opening of the mitochondrial permeability transition pore (mPTP), which triggers cell death [12,13].

The generation of reactive oxygen species (ROS) is an essential part of redox signaling, which in turn affects Ca^2+^ homeostasis in physiology and pathology [14]. Thus, mitochondrial Ca^2+^ uptake activates the electron transport chain of mitochondria that may increase ROS production in this organelle [15]. One of the major ROS producers in the cell, the enzyme NADPH oxidase, is linked to the activity of the glutamate receptor in neurons and to the ATP-dependent Ca^2+^ signaling in astrocytes [16,17]. Mitochondrial- or monoamine oxidase-produced ROS also can activate physiological Ca^2+^ signaling [18,19].

To ensure that ROS are maintained at concentrations that allow cell signaling, but do not become detrimental, cells are equipped with robust antioxidant systems. One such system is controlled by Nrf2, an inducible transcription factor that controls the expression of genes encoding proteins that protect against oxidative, electrophilic, inflammatory and metabolic stress. Widely accepted as an “antioxidant factor”, Nrf2 controls multiple cellular processes, including the biosynthesis of the intracellular antioxidant reduced glutathione (GSH), and the regulation of energy and ROS production by mitochondria and NADPH oxidases [[20], [21], [22]]. Under homeostatic conditions the levels of Nrf2 are maintained low due to constitutive ubiquitination and proteasomal degradation, which is principally mediated by the E3 ubiquitin ligase CRL3^Keap1^ [20]. Electrophiles, such as RTA-408 (omaveloxolone), a drug used for the treatment of Friedreich's ataxia [23], inactivate Keap1 by covalently binding to C151, resulting in Nrf2 activation [24].

Considering the effect of Nrf2 activation on redox balance and energy metabolism – processes which are also Ca^2+^ dependent – we hypothesized that Nrf2 and Ca^2+^ signaling functionally interact. To test this hypothesis, we used primary neuro-glial co-cultures from wild-type (WT), Nrf2-knockout (Nrf2-KO) and Keap1-knockdown (Keap1-KD) mice, and SH-SY5Y cells investigated the effect of activation or inhibition of Nrf2 on cytosolic and mitochondrial Ca^2+^ signaling. We found that Nrf2 deficiency inhibits mitochondrial Ca^2+^ uptake and mitochondrial Ca^2+^ efflux, and substantially reduces mitochondrial Ca^2+^ capacity. Conversely, the activation of Nrf2 by Keap1 deficiency or RTA-408 supplementation accelerate mitochondrial Ca^2+^ efflux, increases mitochondrial Ca^2+^ capacity and protects against mPTP opening.

## 2 Methods

**Primary cell cultures preparation**. Primary mixed cortical cultures were prepared from WT, Nrf2-KO, and Keap1-KD neonatal mice (P1–P3) following UK (1986 Act) and EU (2010/63/EU) ethical guidelines, as previously described [25] with minor modifications. Nrf2-KO mice express a transcriptionally inactive form of Nrf2, in which *LacZ* has been knocked into the C-terminal part of the *Nfe2l2* (the gene encoding Nrf2) locus, resulting in a Nrf2d-lacZ fusion protein devoid of transcriptional activity [26]. Because complete Keap1-KO results in supraphysiological Nrf2 levels and early postnatal lethality [27], we used Keap1-KD mice. These animals carry two floxed alleles of the *Keap1* gene, which reduces its expression and consequently increases the levels of Nrf2 to levels comparable to those that are achievable by pharmacological means [28,29]. All mouse lines were on the C57BL6J genetic background. Cortices were dissected in ice-cold PBS solution (Ca^2+^-, Mg^2+^-free, Gibco-Invitrogen, Paisley, UK, enzymatically dissociated minced, and enzymatically dissociated with 0.05% trypsin (15 min at 37 °C) and resuspended in Neurobasal A media (Gibco-Invitrogen). Cells were plated on poly-l-lysine-coated glass coverslips and maintained at 37 °C (5% CO_2_) in Neurobasal A supplemented with B27, Glutamax, and antibiotics. Following a partial media exchange after 7 days, experiments were conducted at 12–15 DIV. Neurons were identified by their phase-bright, rounded morphology and presence above the glial monolayer. Across all three genotypes, cultures exhibited consistent cell density and morphology.

**SH-SY5Y cell cultivation.** Human neuroblastoma SH-SY5Y cells were cultured in DMEM/F-12 (1:1) supplemented with 10% fetal bovine serum (FBS), 1% penicillin/streptomycin, and 2 mM l-glutamine at 37 °C in a humidified atmosphere containing 5% CO_2_. For experiments, cells were plated either on round coverslips (25 mm diameter) coated with poly-d-lysine to facilitate cell attachment, or in 35 mm Nunc™ cell culture/Petri dishes (Thermo Scientific, cat. No. 150318). Experiments were performed after 2-3 days of cultivation, when cells reached 70–80% confluency.

### 2.1 Imaging of intracellular and mitochondrial calcium concentration

For intracellular and mitochondrial calcium concertation measurement, cells were loaded with 5 μM Fluo- 4 a.m. or Rhod-2, AM, and 0.005% Pluronic in a HEPES buffered salt solution composed of (in mM) 156 NaCl, 3 KCl, 2 MgSO_4_, 1.25 KH_2_PO_4_, 2 CaCl_2_, 10 glucose and 10 HEPES. pH was adjusted to 7.35 with NaOH. Cells were loaded in the dark at room temperature for 40 min and washed with HBSS prior to the experiment.

MitoGCaMP constructs were transfected into primary cultures using Effectene as was described in Ref. [30]. Fluo-4 and MitoGCaMP fluorescence were excited using a 488 nm laser and collected at 500–530 nm. Rhod-2 fluorescence was excited with a 543 nm laser and detected using a 560 nm long-pass filter. Imaging was performed on a ZEISS LSM 980 confocal microscope using 20× or 40× objectives. Data acquisition and analysis were carried out using ZEISS ZEN software.

**Registration of Mitochondrial Calcium Retention Capacity (CRC).** Mitochondrial CRC was measured in permeabilized cells and isolated mitochondria.

To measure ***mitochondrial CRC,*** cells were co-loaded with 3 μM CoroNa™ Green, AM (Invitrogen) + 3 μM X-Rhod-1, AM (Invitrogen) or 5 μM Fluo- 4 a.m. for 40 min at room temperature in HBSS (in mM) 156 NaCl, 3 KCl, 2 MgSO_4_, 1.25 KH_2_PO_4_, 2 CaCl_2_, 10 glucose and 10 HEPES, pH was adjusted to 7.35 with NaOH. Afterwards cells were placed under the confocal microscope Zeiss 900 LSM or ZEISS LSM 980 with 40× objective and fluorescence were measured in two channels 488/516–530 nm excitation/emission for CoroNa™ Green or Fluo-4 and 543/560-600 nm nm excitation/emission for X-Rhod-1, AM. For plasma membrane permeabilization HBSS solution was replaced with “pseudo-intracellular” solution 1 (135 mM KCl, 10 mM NaCl, 20 mM HEPES, 5 mM pyruvate, 5 mM malate, 0.5 mM KH_2_PO_4_, 1 mM MgCl_2_, 5 mM EGTA, and 1.86 mM CaCl_2_ (to yield a free [Ca^2+^] of ∼100 nM), pH 7.1 + 20 μM digitonin) as was described in Ref. [31]. Evaluation of plasma membrane permeabilization was monitored by egress of CoroNa™ Green from the cytosol, and strong co-localization of the X-Rhod-1 signal with the mitochondria (Fig. 4 A 1 and 2). Experiments with Fluo-4 were performed without CoroNa™ Green and permeabilization was monitored by egress of Fluo-4 from the cytosol, and co-localization of the Fluo-4 signal with the mitochondria. Usually, 2-3 min was enough for permeabilization process. After permeabilization the digitonin was washed out by replacing (3 times) the buffer with the “pseudo-intracellular” solution. Before the start of the titration “pseudo-intracellular” solution was replaced with “pseudo-intracellular” solution without CaCl_2_ and EGTA. CRC was evaluated by sequential addition of 10 μM CaCl_2_ and monitoring the level of mitochondrial calcium (X-Rhod-1 or Fluo-4). To monitor mitochondrial calcium, up to 10 regions of interest within the cell were selected in areas where a clearly defined mitochondrial network was observed. On each experimental day, CRC was assessed in control cells and in cells treated with RTA-408. For each day, the CRC value in control cells was taken as 100%, and the effect of RTA-408 was evaluated relative to the control on that same experimental day. Calcium efflux was calculated using the linear fit function in OriginPro software. For the calculations, Ca^2+^ efflux was evaluated during the first two calcium additions, which caused a sharp increase in mitochondrial calcium followed by calcium efflux.

***Evaluation of CRC in isolated mitochondria***. Mitochondria were isolated from the liver by differential centrifugation as described in Ref. [32]. CRC assessment was performed using the method of mitochondrial calcium release, using cell impermeant calcium-sensitive fluorescent dye - Calcium Green 5 N (0.5 μM; excitation 506 nm and emission 532 nm). Experiments were performed on CLARIOstar Plus multi-mode microplate reader (BMG LABTECH) in incubation media (KCl 120 mM, Tris-base 10 mM, KH_2_PO_4_ 1 mM, MgSO_4_ 1 mM, glutamate 5 mM malate 1 mM, pH was adjusted to 7.1 with KOH) as was described in Ref. [33]. CRC of control samples was taken as 100% and the changes of CRC in different experimental conditions were calculated against control (100%). Mitochondria were added in the final concentration 0.5 mg/ml, evaluated in terms of protein concentration. The protein content was measured by the Bradford test with BSA as the standard.

### 2.2 Registration of PTP in intact cells

For registration of PTP activation, SH-SY5Y cells were loaded with 25 nM TMRM (Invitrogen) for 40 min at room temperature. PTP activation was measured as a drop of mitochondrial membrane potential after application of 25 μM of ferutinin [12,34]. TMRM fluorescence was measured using CELENA® S Digital Imaging System (Logos Biosystems) with 20× objective using RFP filter (TMRM - 552_excitation_/574_emission_ nm). Images were collected at intervals of 20 s and minimum illumination to reduce phototoxicity.

### 2.3 Statistics

All values are expressed as mean ± SEM. Differences between means were analyzed using the Student's t-test, one-way or two-way analysis of variance (ANOVA) depending on the number of groups and variables in each experiment. Data was then submitted to Tukey or Bonferroni post hoc test using Origin Pro software. The null hypothesis was rejected when the P value was <0.05. The choice of statistical test has been stated in each figure legend.

### 2.4 Data availability

Data that support the findings in this study are available from the corresponding author upon reasonable request.

## 3 Results

### 3.1 Nrf2 deficiency increases physiological Ca^2+^ signaling in neurons and astrocytes

**Fig. 1:**
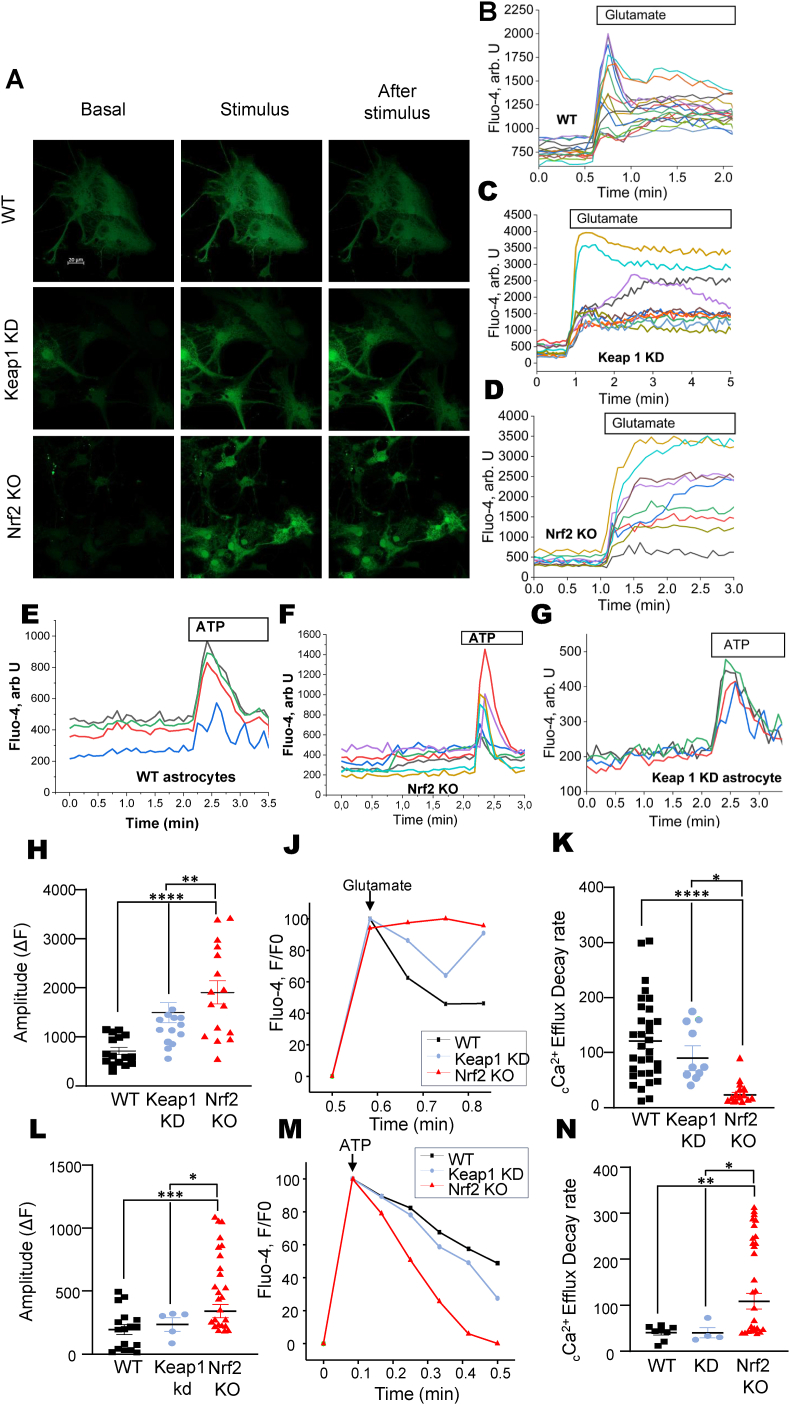
**Nrf2 deficiency differentially modulates calcium signaling dynamics in primary cortical neurons and astrocytes. A**. Representative confocal imaging of primary astrocytes. Astrocytes loaded with the fluorescent Ca^2+^ indicator Fluo-4 are shown at three experimental stages: Basal (resting state), Stimulus (peak response during 100 μM ATP application), and After stimulus (washout phase). Images illustrate the different fluorescence intensities across wild-type (WT), Keap1 kd, and Nrf2 KO genotypes. Representative experimental traces in mixed cultures. Traces of Fluo-4 fluorescence intensity (F/F_0_) show the typical [Ca^2+^]_c_ response to 5 μM Glutamate (**B, C, D**) and 100 μM ATP (**E, F, G**) in WT, Keap1 kd, and Nrf2 KO cultures. Gray bars indicate the duration of activator application. **H**. Normalized recovery kinetics. Comparative normalized traces (F/F_0_) aligned at the peak of stimulation for 5 μM Glutamate. Nrf2 deficiency (red line) significantly alters the recovery profile, showing a sustained signal in neurons and an accelerated decay in astrocytes compared to WT and Keap1 kd groups. **J**. Neuronal Amplitude. Statistical comparison of the peak amplitude (ΔF) of glutamate-induced [Ca^2+^]_c_ rises in neurons. Nrf2 KO neurons exhibit a significantly higher amplitude compared to WT (∗∗∗∗p ≤ 0.0001) and Keap1 kd (∗∗p ≤ 0.01). **K**. Neuronal [Ca^2+^] Efflux Decay Rate. Quantification of calcium clearance efficiency in neurons. Nrf2 deficiency significantly reduces the efflux decay rate compared to WT (∗∗∗∗p ≤ 0.0001) and Keap1 kd (∗p ≤ 0.05), indicating impaired recovery kinetics. **L**. Representative traces of [Ca^2+^]_c_ transients (F/F_0_) focused on the ATP-induced response in astrocytes for WT, Keap1 kd, and Nrf2 KO genotypes. **M**. Statistical comparison of the maximum amplitude (ΔF) of the ATP response in astrocytes. Nrf2 deficient astrocytes show an increased calcium peak compared to WT (∗∗∗p ≤ 0.001) and Keap1 kd (∗p ≤ 0.05). **N.** Astrocytic [Ca^2+^]_c_ Efflux Decay Rate. Quantification of the recovery rate in astrocytes. In contrast to neurons, Nrf2 KO astrocytes exhibit an accelerated efflux decay rate compared to WT (∗∗p ≤ 0.01) and Keap1 kd (∗p ≤ 0.05). Data are presented as mean ± SEM. Statistical analysis was performed using one-way ANOVA followed by Dunnett's multiple comparisons test. For each experiment, a minimum of 4 cells per coverslip were analyzed across independent cell passages.

Glutamate and ATP are the most common physiological activators of Ca^2+^ signaling in neurons and astrocytes. To assess the effect of Nrf2 on Ca^2+^ signaling in primary neurons and astrocytes, we loaded cells with the fluorescent Ca^2+^ indicator fluo-4 (Fig. 1A). Application of 5 μM glutamate induced the typical for cortical neurons rise in cytosolic Ca^2+^ concentration ([Ca^2+^]_c_) in cultures from WT mice (Fig. 1A and B). Nrf2 deficiency significantly increased the amplitude of glutamate-induced Ca^2+^ signal, whereas the response to glutamate in Keap1-KD neurons was close to that of WT cells (Fig. 1A, B, C, D, H). Notably, the rate of recovery of [Ca^2+^]_c_ of Nrf2-KO neurons was significantly slower compared to WT or Keap1-KD neurons (Fig. 1J and K).

The amplitude of 100 μM ATP-induced [Ca^2+^]_c_ rise in astrocytes from Nrf2-deficient mice was also higher that in WT and Keap1-deficient astrocytes (Fig. 1 E, F, G, L). By contrast, the rate of [Ca^2+^]_c_ recovery after ATP application was similar in WT, Keap1-KD and Nrf2-KO neurons (Fig. 1M and N).

Thus, Nrf2 deficiency increases Ca^2+^ signaling by different mechanisms in neurons and astrocytes. One possible explanation for this effect could be lower mitochondrial Ca^2+^ uptake in Nrf2-KO cells due to lower mitochondrial membrane potential [21,35].

### 3.2 Nrf2 alters mitochondrial Ca^2+^ handling in response to physiological activators in neurons and astrocytes

**Fig. 2:**
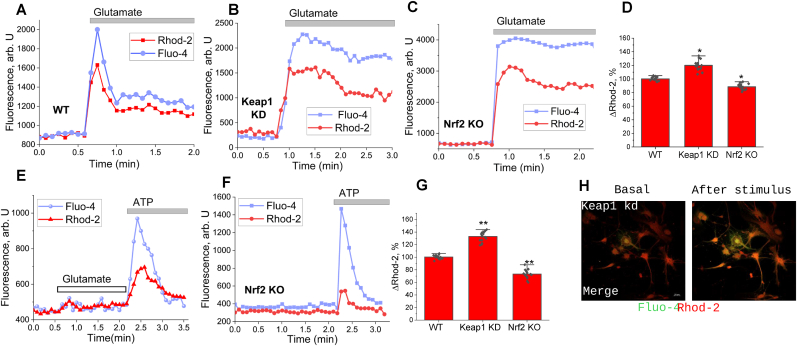
**Nrf2 regulates mitochondrial Ca^2+^ uptake in neurons and astrocytes.** Representative traces from neurons and astrocytes co-loaded with Fluo-4 (green, cytosolic Ca^2+^) and Rhod-2 (red, mitochondrial Ca^2+^) showing fluorescence intensity over time following stimulation with 5 μM glutamate and 100 μM ATP, respectively. Gray bars indicate the duration of glutamate or ATP application. **A.** Representative trace of WT neurons after glutamate stimulation. **B.** Representative traces of Keap1 kd neurons after glutamate stimulation. **C.** Representative traces Nrf2 KO neurons after glutamate stimulation. **D.** Amplitude of mitochondrial Ca^2+^ signal in response glutamate. WT was taken as 100% **E**. Representative traces of WT astrocytes after ATP stimulation. **F.** Representative traces of Nrf2 KO astrocytes after ATP stimulation. **F.** Amplitude of mitochondrial Ca^2+^ signal in response 100 μM ATP. WT was taken as 100%**.** Representative confocal imaging of Keap1 kd primary astrocytes. Astrocytes co-loaded with the fluorescent Ca^2+^ indicators Fluo-4 (green) and Rhod-2 (red) are shown at two experimental stages: Basal (resting state) and After stimulus (peak response following 100 μM ATP application).

To assess the effect of Nrf2 on mitochondrial Ca^2+^, we used co-loading of neurons and astrocytes with fluo-4 (cytosolic Ca^2+^) and rhod-2 (an indicator which, due to its positive charge, is mainly distributed in mitochondria). In WT neurons, application of 5 μM glutamate induced peak-like increase in the cytosol and Ca^2+^ uptake into mitochondria (Fig. 2A–D). In astrocytes, mitochondrial Ca^2+^ uptake was induced by increase of [Ca^2+^]_c_ in in response to 100 μM ATP (Fig. 2E–H). It should be noted that the mitochondrial Ca^2+^ uptake in response to 5 μM glutamate was increased in Keap1-KD neurons (120 ± 7% of WT, p < 0.05; n = 19), and in response to 100 μM ATP in astrocytes (133 ± 7 of WT, p < 0.01; n = 18; Fig. 2 A, B, D, E, G, H). In Nrf2-KO neurons, mitochondrial Ca^2+^ uptake in response to 5 μM glutamate was 88 ± 4% of WT and lower than in Keap1-KD neurons, although the cytosolic Ca^2+^ signal was higher than in WT neurons (Fig. 2A–C, D). The same correlation was found in Nrf2- KO astrocytes – higher rise of Ca^2+^ in cytosol but lower mitochondrial calcium uptake in response to 100 μM ATP than in WT and Keap1-KD cells (73 ± 7% of WT; p < 0.01; N = 3 experiments, n = 17; Fig. 2 F, G, H).

**Fig. 3:**
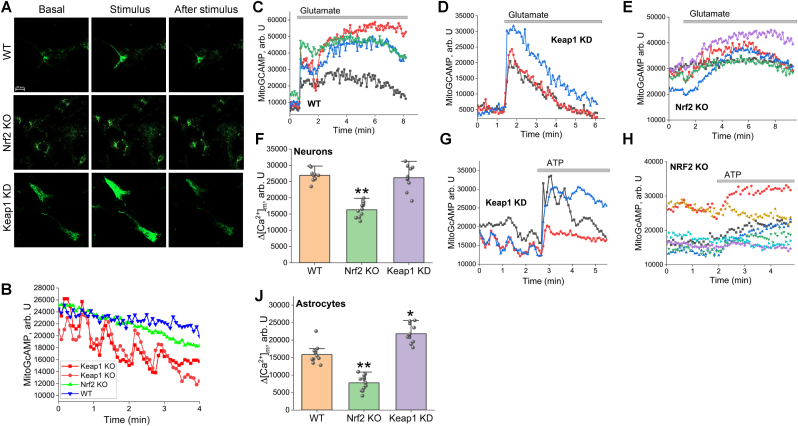
**Nrf2 deficiency impairs mitochondrial calcium uptake in primary neurons and astrocytes. A.** Representative experimental traces of mitochondrial Ca^2+^ dynamics in neurons. The top panels show MitoGCaMP fluorescence intensity (arb U) comparing WT, Nrf2 KO, and Keap1 kd groups, illustrating the overall differences in uptake capacity. **B.** Representative confocal imaging of primary cultures expressing the mitochondrial Ca^2+^ indicator MitoGCaMP. Images show mitochondrial fluorescence at Basal (resting), Stimulus (peak response), and After stimulus (recovery) stages for wild-type (WT), Nrf2 KO, and Keap1 kd genotypes. **C.** Representative experimental traces of the three separate groups. Individual traces for WT, Keap1 kd, and Nrf2 KO neurons show the specific mitochondrial Ca^2+^ response profile for each genotype upon 5 μM Glutamate application. **D.** Statistical analysis of the maximum amplitude of mitochondrial Ca^2+^ rises Δ[Ca^2+^]_m_ in neurons. The bar graph demonstrates that Nrf2 deficiency significantly reduces mitochondrial Ca^2+^uptake compared to WT and Keap1 kd groups (∗∗p ≤ 0.01). **E.** Representative experimental traces in astrocytes. The panels show merged and individual MitoGCaMP responses for Keap1 kd and Nrf2 KO astrocytes during 100 μM ATP stimulation, highlighting the differential mitochondrial sequestration capacity. **F**. Statistical analysis of the maximum amplitude of mitochondrial Ca^2+^ rises Δ[Ca^2+^]_m_ in astrocytes. The bar graph shows a significant decrease in mitochondrial calcium uptake in Nrf2 KO astrocytes (∗∗p ≤ 0.01 vs WT), while Keap1 kd astrocytes exhibit a significantly higher uptake capacity (∗p ≤ 0.01 vs WT). Data are presented as mean ± SEM. Statistical analysis was performed using one-way ANOVA followed by Dunnett's multiple comparisons test. For each experiment, a minimum of 4 cells per coverslip were analyzed.

Considering that part of the rhod-2 fluorescence could be from cytosol we used another mitochondrial indicator for Ca^2+^, a genetically encoded MitoGCaMP (Fig. 3 A). We found that basal [Ca^2+^]_m_ was similar in WT, Nrf2-KO and Keap1-KD cells, but interestingly, the majority of Keap1-KD neurons and astrocytes (∼65%) had a peak like oscillations in MitoGCaMP fluorescence. This type of signal was not observed with cytosolic Ca^2+^ indicators in these cells, suggesting that these oscillations are specific for mitochondria of Keap1-KD cells (Fig. 3B).

**Fig. 4:**
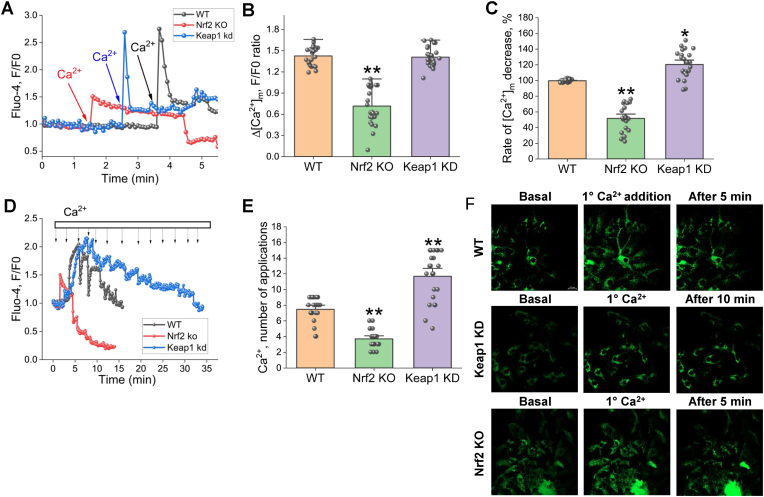
**Nrf2 deficiency decreases mitochondrial Ca^2+^ buffering capacity in neurons and astrocytes.** Measurements of mitochondrial Ca^2+^ capacity were performed in permeabilized neurons and astrocytes loaded with the fluorescent Ca^2+^ indicator Fluo-4. Repeated additions of 5 μM CaCl_2_ were applied to evaluate mitochondrial Ca^2+^ buffering capacity and the susceptibility to mitochondrial permeability transition pore (mPTP) opening. **A.** Representative traces of Fluo-4 fluorescence (F/F_0_) in permeabilized cells during sequential Ca^2+^ additions in WT (black), Keap1 kd (blue) and Nrf2 KO (red), conditions. Arrows indicate individual Ca^2+^ additions. **B.** Cytosolic Ca^2+^ response amplitude. Statistical comparison of the change in fluorescence ratio (Δ[Ca^2+^]c, F/F_0_) following Ca^2+^ application. Nrf2 KO cells show a significantly reduced Ca^2+^ response compared to WT (∗∗p ≤ .). **C.** Rate of cytosolic Ca^2+^ increase. Quantification of the rate of [Ca^2+^]c increase (%) after Ca^2+^ addition. Nrf2 deficiency significantly decreases the rate compared to WT (∗∗p ≤ …), whereas Keap1 kd cells show an increased rate (∗p ≤ …). **D.** Representative traces of mitochondrial Ca^2+^ buffering capacity in permeabilized cells exposed to repeated Ca^2+^ additions. Rapid fluorescence loss reflects mPTP opening. Nrf2 KO cells show earlier fluorescence loss, indicating reduced mitochondrial Ca^2+^ capacity, whereas Keap1 KD cells sustain Ca^2+^ loading for longer periods. **E.** Mitochondrial Ca^2+^ capacity. Statistical comparison of the number of Ca^2+^ additions required to induce mPTP opening. Nrf2 deficiency significantly decreases mitochondrial Ca^2+^ capacity compared to WT (∗∗p ≤ …), while Keap1 kd significantly increases mitochondrial Ca^2+^ capacity (∗∗p ≤ …). **F.** Representative confocal images of cells loaded with Fluo-4 showing mitochondrial Ca^2+^ responses at different experimental stages: Basal, first Ca^2+^ addition, and later time points following repeated Ca^2+^ loading in WT, Keap1 kd, and Nrf2 KO cells. Data are presented as mean ± SEM. Statistical analysis was performed using one-way ANOVA followed by Dunnett's multiple comparisons test. For each experiment, a minimum of 4 cells per coverslip were analyzed across independent cell passages.

In agreement with the data obtained with the rhod-2 indicator, the increase of MitoGCaMP fluorescence in neurons and astrocytes in response to 5 μM glutamate (neurons) and 100 μM ATP (astrocytes) was lower in Nrf2-KO compared to WT and Keap1-KD cells (Fig. 3C, D, F, H, J).

It should be also noted that the mitochondrial Ca^2+^ signal was not only different among the genotypes by amplitude, but also by the shape of the signal (Fig. 3C–H). Thus, the rate of Ca^2+^ uptake into mitochondria in Nrf2-KO neurons was slower compared to WT or Keap1-KD neurons (Fig. 3E). The rate of mitochondrial Ca^2+^ efflux was also slower in Nrf2-KO and Keap1-KD neurons compared to WT. Thus, in intact cells Nrf2 deficiency leads to a decrease in mitochondrial Ca^2+^ uptake and slower mitochondrial Ca^2+^ efflux in neurons and astrocytes. However, the amplitude and shape of the mitochondrial Ca^2+^ signal may reflect differences in physiological responses to stimulus. To avoid this complexity, next we applied Ca^2+^ to permeabilized cells in pseudo-intracellular medium.

### 3.3 Lack of Nrf2 decreases mitochondrial Ca^2+^ uptake in permeabilized neurons and astrocytes

Application of 3 μM CaCl_2_ to permeabilized primary neurons and astrocytes loaded with the Ca^2+^ indicator fluo-4 induced Ca^2+^ uptake by mitochondria (Fig. 4A, B, F) followed by recovery of the signal – Ca^2+^ efflux as a result of the function of mitochondrial Na^+^/Ca^2+^ exchanger. It should be noted that the rate of mitochondrial Ca^2+^ efflux was also much lower in Nrf2-KO compared to WT cells (Fig. 4A–C). Keap1-KD cells had higher activity of mitochondrial Na^+^/Ca^2+^ exchanger and faster efflux of Ca^2+^ from mitochondria, although the amplitude of [Ca^2+^]_c_ in response to 3 μM CaCl_2_ was similar to WT cells (Fig. 4 A, B, C, F). Together, these results suggest that Nrf2 alters the mitochondrial Ca^2+^ handling and facilitates the removal of Ca^2+^ from mitochondria.

### 3.4 Nrf2 deficiency decreases mitochondrial Ca^2+^ capacity

The mitochondria serve as a short-term Ca^2+^ buffer in the cell. However, changes in mitochondrial Ca^2+^ handling, or intracellular Ca^2+^ deregulation, may lead to mitochondrial Ca^2+^ overload exceeding the Ca^2+^ buffering capacity and triggering opening of the mPTP, ultimately causing cell death [36]. Using permeabilized neurons and astrocytes loaded with fluo-4, we subsequently applied 5 μM CaCl_2_ until fast loss of the fluorescence from single mitochondria [31,37]. We found that the mitochondrial Ca^2+^ capacity (the number of Ca^2+^ applications needed to fast and transient loss of the signal in Nrf2-KO neurons and astrocytes was significantly lower compared to WT (Fig. 4 D, E, F). Conversely, the mitochondrial Ca^2+^ capacity was higher in permeabilized Keap1-KD primary neurons and astrocytes (Fig. 4 D, E, F).

### 3.5 The pharmacological Nrf2 activator RTA-408 increases mitochondrial Ca^2+^capacity

**Fig. 5:**
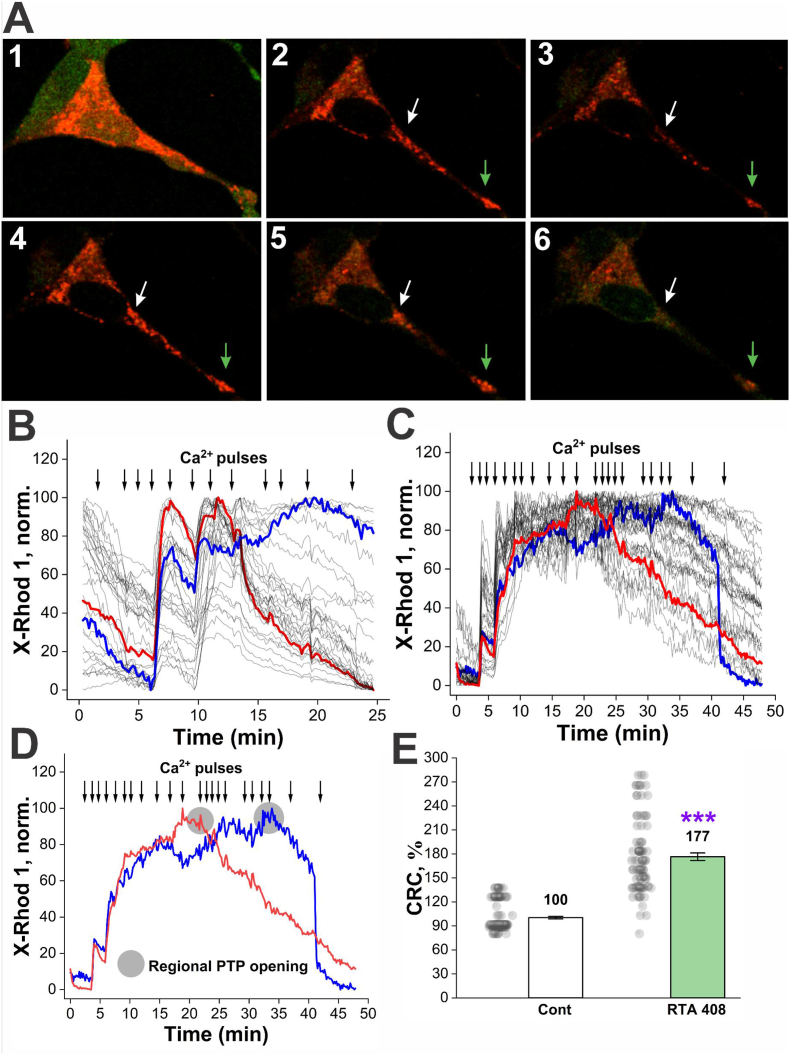
**Evaluation of mitochondrial CRC in permeabilized SH-SY5Y cells. A.** Representative images of a single cell at different time points: **1.** a non-permeabilized cell loaded with CoroNa™ Green and X-Rhod-1; **2.** a permeabilized cell after the first calcium pulse, showing intense X-Rhod-1 fluorescence, with the mitochondrial network exhibiting a well-defined, branched morphology; **3.** a decrease in X-Rhod-1 signal corresponding to calcium efflux, while the mitochondrial network retains its normal branched structure and remains responsive to subsequent calcium additions; **4.** a subsequent calcium pulse, with the mitochondrial network still displaying an intact branched morphology; **5.** onset of mitochondrial rounding and swelling, accompanied by a decline in X-Rhod-1 fluorescence and loss of responsiveness to further calcium additions, indicative of PTP opening; **6.** near-complete loss of X-Rhod-1 signal. **B.** Representative experimental trace from control cells; **C.** Representative experimental trace from RTA-408–treated cells. **D.** Representative cellular regions in which PTP opening was detected; gray circles indicate time points interpreted as PTP opening events (taken from **C**); **E.** Statistical comparison of mitochondrial CRC values between control and RTA-408–treated groups. ∗∗∗p ≤ 0.001, two-sample *t*-test. Experiments were performed using three independent cell passages. N = 6, n = 125 for control; N = 6, n = 96 for RTA-408, where **N** denotes the number of coverslips (Petri dishes) and **n** denotes the number of analyzed cells.

**Fig. 6:**
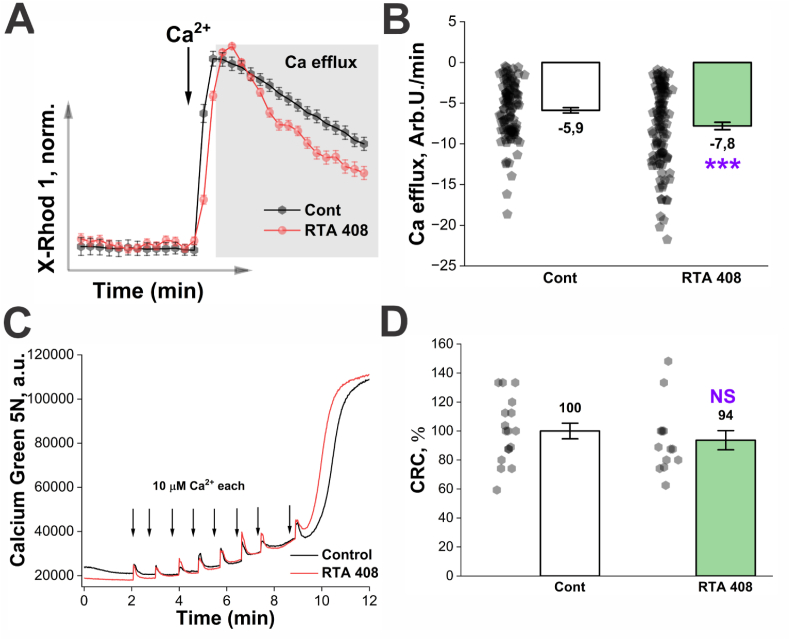
**RTA-408 treatment enhances mitochondrial calcium efflux.** A. Averaged X-Rhod-1 fluorescence signal from all cells within a single Petri dish, shown as a representative recording. The rate of calcium efflux was quantified as the decline in X-Rhod-1 fluorescence following a calcium pulse, indicated by the blue rectangle. B. Statistical comparison of calcium efflux rates between control cells and RTA-408–treated cells. ∗∗∗p ≤ 0.001, two-sample *t*-test; the number of experiments and analyzed cells corresponds to those reported in the previous figure. C. Representative recording from experiments assessing the direct effect of RTA-408 on the CRC of isolated mitochondria. **D.** Statistical analysis of CRC in mitochondria from control and RTA-408–treated samples. NS, not significant, two-sample *t*-test. Mitochondria were isolated from three independent animals; n = 17 for control samples and n = 13 for RTA-408–treated samples.

To evaluate the effect of pharmacological activation of Nrf2 on mitochondrial Ca^2+^ retention capacity (CRC), we used the potent inducer RTA-408. To avoid any effect of interaction of neurons and astrocytes and also the difference in physiological responses shown in Fig. 1, Fig. 2, Fig. 3, we used homogeneous cell culture neuroblastoma SH-SY5Y cells, which were treated with 15 nM RTA-408 for 24 h. Mitochondrial CRC was subsequently assessed in permeabilized cells by sequential titration with 10 μM CaCl_2_. The changes in mitochondrial Ca^2+^ levels were monitored using the fluorescent indicator X-Rhod-1. The initial Ca^2+^ addition did not induce a pronounced increase in mitochondrial Ca^2+^, most likely due to the presence of residual EGTA in the medium (Fig. 5B and C). However, following the second to third Ca^2+^ addition, a sharp increase in matrix Ca^2+^ concentration was observed, as reflected by a robust rise in X-Rhod-1 fluorescence intensity (Fig. 5 A, panels 2-4; Fig. 5B and C). This phase was subsequently followed by a decline in X-Rhod-1 fluorescence, which we interpreted as Ca^2+^ efflux from the mitochondria (Fig. 5 A, panels 2-4; Fig. 5B–D). In regions where the mitochondrial Ca^2+^ levels approached the threshold, mitochondria underwent a characteristic rounding and swelling (Fig. 5 A-5), accompanied by a loss of X-Rhod-1 signal (Figs. 5 A–6), as well as absence of a response to subsequent Ca^2+^ pulses (Fig. 5 D). Notably, RTA-408 treatment resulted in a 1.8-fold increase in mitochondrial CRC compared with vehicle-treated control cells (Fig. 5C, D, E).

The observed increase in CRC could be attributed either to direct inhibition of the mPTP or to enhanced mitochondrial Ca^2+^ efflux, which in neuronal cells, is predominantly mediated by the mitochondrial Na^+^/Ca^2+^/Li^+^ exchanger (NCLX). To address this possibility, we compared calcium efflux kinetics in vehicle-treated control cells and cells treated with RTA-408. Quantitative analysis revealed that RTA-408 treatment increased the rate of Ca^2+^ efflux by an average of 1.3-fold relative to vehicle-treated cells (Fig. 6A and B).

### 3.6 Application of pharmacological Nrf2 activator RTA-408 to isolated mitochondria does not change the function of mPTP

To determine whether RTA-408 directly modulates PTP activation, we next examined its effect on CRC in isolated mitochondria. Preincubation of isolated mitochondria with 15 nM RTA-408 for 10 min prior to Ca^2+^ titration did not significantly affect either the rate of mitochondrial calcium uptake or CRC (Fig. 6C and D), indicating that RTA-408 does not act directly on the mitochondrial PTP machinery.

**Fig. 7:**
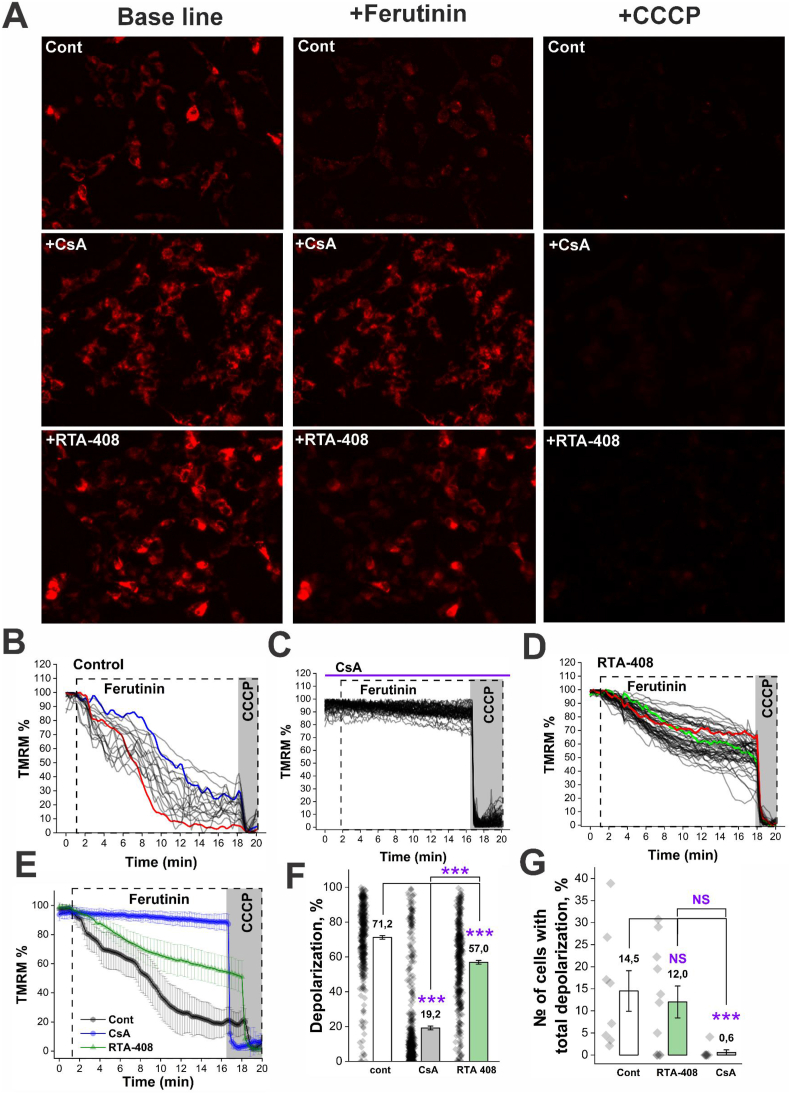
**RTA-408 treatment attenuates PTP opening in intact cells. A.** Representative images of TMRM-loaded cells are shown for the control group (top horizontal row), CsA-treated cells (middle horizontal row), and RTA-408–treated cells (bottom horizontal row). For each experimental condition, images acquired at baseline - prior to ferutinin application (1st – 2nd min of the experiment) - are shown in the left vertical column. Images illustrating the effect of ferutinin, recorded immediately before CCCP addition (17th −18th min of the experiment), are shown in the middle vertical column, while images reflecting the effect of CCCP are shown in the right vertical column. Representative experimental traces from control cells (**B**), CsA-treated cells (**C**), and RTA-408–treated cells (**D**). **E.** Comparison of the kinetics of the ferutinin-induced effect across the different experimental groups, shown as averaged data from cells within a single representative Petri dish. **F.** Statistical comparison of the magnitude of the effect among the three experimental groups. ∗∗∗p ≤ 0.001, one-way ANOVA with post hoc Tukey test. Experiments were performed using three independent cell passages. Control: N = 6, n = 400; CsA: N = 7, n = 548; RTA-408: N = 7, n = 440, where N denotes the number of coverslips and n denotes the number of analyzed cells. Each dot in the distribution represents an individual cell. **G.** Statistical comparison among the three groups based on the proportion of cells exhibiting complete depolarization. Each dot represents the percentage of fully depolarized cells within an individual coverslip.

In the next series of experiments, mPTP opening was induced in intact cells using ferutinin, a natural phytoestrogen isolated from plants of the Ferula genus. Ferutinin has been previously shown to function as a Ca^2+^ ionophore, and at specific concentrations can induce mitochondrial Ca^2+^ overload, mPTP opening, and cell death [12,30]. SH-SY5Y cells were treated with 15 nM RTA-408 for 24 h prior to ferutinin exposure. Application of 25 μM ferutinin caused a gradual dissipation of the mitochondrial membrane potential (Ψ_M_), detected as a progressive decline in TMRM fluorescence over a 15-min period following ferutinin addition (Fig. 7A and B). At the end of each experiment, 1 μM CCCP was applied to fully collapse Ψ_M_, allowing normalization of the TMRM signal (Fig. 7A and B).

In control cells, ferutinin induced an average mitochondrial depolarization of 71.2% during the 15-min recording period. Moreover, approximately 15% of the cells exhibited complete depolarization, manifested as a total loss of TMRM fluorescence and absence of response to subsequent CCCP application (Fig. 7 A, B, F, G). To confirm that the observed loss of Ψ_M_ was specifically associated with mPTP opening, cells were pretreated with the classical mPTP inhibitor cyclosporin A (CsA; 1 μM, 10 min). Under these conditions, ferutinin induced only a 19% decrease in Ψ_M_ (Fig. 7A–C, F). Importantly, CsA completely abolished the effect of ferutinin in the majority of cells (Fig. 7 F; see single-cell response distributions) and markedly reduced the proportion of cells undergoing complete depolarization, to near zero (Fig. 7 G).

Treatment with RTA-408 significantly attenuated the effect of ferutinin, reducing the average depolarization from 71.2% in vehicle-treated control cells to 57% in RTA-408–treated cells; however, this protective effect was less pronounced than that observed with CsA (Fig. 7A–D–F). While ferutinin consistently induced complete depolarization in a subset of control cells across all experiments, some RTA-408–treated dishes contained no fully depolarized cells, reducing the overall proportion of cells exhibiting complete depolarization to 12%. Nevertheless, the differences between control and RTA-408–treated groups did not reach statistical significance for this parameter (Fig. 7A–D–F).

Taking together, these data demonstrate that RTA-408 treatment significantly inhibits mitochondrial PTP opening in intact cells, an effect that is likely mediated by enhanced mitochondrial Ca^2+^ efflux rather than direct inhibition of the mPTP. Consistent with this interpretation, experiments using isolated mitochondria indicate that RTA-408 does not directly modulate mitochondrial CRC, suggesting that the observed cellular effects are most likely mediated through activation of Nrf2.

## 4 Discussion

Ca^2+^ signaling is universal for all cell types and is implicated in almost all biological processes. Here, we show that the antioxidant transcription factor Nrf2, which controls 1% of genome, also affects Ca^2+^ signaling. We used the most common Ca^2+^ triggers in neurons and astrocytes – glutamate and ATP - to study the effect of Nrf2 on Ca^2+^ signaling in these cells. Our results indicate that both – activation of Nrf2 by Keap1 knockdown or Nrf2 deficiency - lead to increased glutamate- or ATP- induced Ca^2+^ signal. It should be noted that glutamate and ATP induce a [Ca^2+^]_c_ rise by different mechanisms. In neurons, glutamate activates opening of the plasmalemmal Ca^2+^ channel, whereas ATP activates metabotropic P2Y receptors and release of Ca^2+^ from the endoplasmic reticulum [14].This suggests that the observed Nrf2-induced changes in Ca^2+^ signaling are not due to receptor specific mechanism(s). This conclusion is further supported by the absence of any differences in the amplitude or oscillations frequency in NMDA receptor activation in seizure-like activity between control neurons and neurons in which Nrf2 was pharmacologically activated [24]. One possible explanation of these results may be oxidation of lipids for ATP-induced calcium signaling in astrocytes [19] or oxidative alteration of glutamate receptors or transporters [38]. Difference in buffering Ca^2+^ capacity of mitochondria due to differences in mitochondrial membrane potential [21,35] also could be one of the factors contributing to the Nrf2-induced changes in amplitude of Ca^2+^ signal in response to physiological activators. The lower rate of Ca^2+^ efflux from neurons potentially could be explained by lower expression of sarco-endoplasmic reticulum Ca^2+^-ATPase (SERCA) in Nrf2 deficiency [39]. Interestingly, pharmacological activation of Nrf2 by dimethyl fumarate also decreased SERCA expression [40], and could be one of the explanations for the lower rate of Ca^2+^ efflux in Keap1-KD astrocytes.

We found here that the deficiency in Nrf2 leads to a significant decrease in the amplitude of mitochondrial Ca^2+^ uptake in intact neurons and astrocytes. Experiments in intact cells using MitoGCaMP showed that compared to WT, the rate of mitochondrial Ca^2+^ uptake is lower in Nrf2-KO neurons and astrocytes, suggesting alteration of mitochondrial Ca^2+^ uniporter, although the rate of Ca^2+^ uptake was similar in permeabilised cells. Activation of Nrf2 genetically (by Keap1-KD) or pharmacologically (by RTA-408) did not affect the amplitude or the rate of mitochondrial Ca^2+^ uptake despite the higher mitochondrial membrane potential in these cells [21] and higher expression of mitochondrial Ca^2+^ uniporter in Nrf2 activated cells [41]). One important finding is that Nrf2 deficiency resulted in a significantly slower mitochondrial Ca^2+^ efflux and conversely, pharmacological or genetic activation of Nrf2 significantly activated this process. The role of mitochondrial Na^+^/Ca^2+^ exchanger (NCLX) in the generation of ROS in the electron transport chain has been shown by a number of studies [2,42,43]. Less is known about the effect of ROS on the activity of NCLX. The inhibition of mitochondrial Ca^2+^ efflux in Nrf2-KO cells could potentially be due to oxidation of this transporter. However, the stimulation of NCLX function by pharmacological or genetic activation of Nrf2 is likely redox independent, because Keap1-KD neurons and astrocytes produce more ROS in mitochondria compared to WT cells [22]. One of the possible ways of activation of NCLX in Keap1-KD cells could be through protein kinase A (PKA), which is involved in the regulation of NCLX [44]. Neurodegenerative disorders such a Parkinson's or Alzheimer's disease are associated with inhibition of NCLX function [[45], [46], [47], [48]] and the protective effect of Nrf2 activation in these diseases could be partially explained by NCLX re-activation. Notably, the levels of NCLX are also lower in frataxin-deficient cardiomyocytes and dorsal root ganglia neurons, leading to mitochondrial Ca^2+^ overload, mitochondrial swelling, and apoptosis [49]. We speculate that the ability of Nrf2 to modulate Ca^2+^ signalling could be one of the critical mechanisms by which RTA-408 exerts its beneficial effects in Friedreich's ataxia patients [50].

The higher mitochondrial Ca^2+^ exchange in resting Keap1-KD neurons and astrocytes may explain the previously reported higher rate of mitochondrial bioenergetics and faster consumption of mitochondrial substrates ([21,51]. The increased mitochondrial Ca^2+^ capacity and the higher threshold of opening of the mPTP in Keap1-KD cells, and in mitochondria of cells with pharmacological activation of Nrf2, could be explained by the higher rate of mitochondrial Ca^2+^ efflux in these cells or/and the antioxidant effects of Nrf2. This is in agreement with studies showing that administration of the Nrf2 activator sulforaphane to rats increases the antioxidant defences and inhibits redox-sensitive mPTP opening in isolated mitochondria from brain and liver [52]).

An additional plausible explanation for the these effects may involve alterations in the expression of proteins that directly or indirectly regulate mitochondrial calcium transport. Genetic manipulations of the Keap1/Nrf2 system have been extensively studied, and numerous RNA-seq datasets from Nrf2-or Keap1-deficient models, including brain-derived cell types are now available in public repositories or from lead authors [[53], [54], [55]]. However, in most studies on Keap1/Nrf2-dependent transcriptional changes the main focus was on genes involved in antioxidant defense, detoxification, metabolic regulation, and pro-/anti-inflammatory signaling, rather than on specific components of mitochondrial calcium handling. To date, there is no direct evidence linking Keap1/Nrf2 perturbations to transcriptional changes in core components of mitochondrial Ca^2+^ uptake and efflux, such as MCU, MICU1/2, EMRE, TMEM65 or NCLX. Nevertheless, given the central role of Nrf2 in redox homeostasis and the tight coupling between redox state and mitochondrial Ca^2+^ dynamics, such regulation remains mechanistically plausible. In this context, a systematic re-analysis of existing Nrf2/Keap1 transcriptomic datasets with a specific focus on genes involved in mitochondrial Ca^2+^ transport may provide important insights in Nrf2 functioning. Considering possible role in protein oxidation the redox proteomix studies of Nrf2 ko and Keap1 kd mice could be beneficial for understanding the changes in calcium signaling and it could be a subject for separate study.

In conclusion, Nrf2 modulates Ca^2+^ signalling in neurons and astrocytes and activates mitochondrial Ca^2+^ transport, adding another layer to the multifaceted mechanisms by which this transcription factor contributes to the defence in these cells.

## CRediT authorship contribution statement

**Alessandra Preziuso:** Investigation, Methodology, Writing – original draft. **Artyom Y. Baev:** Formal analysis, Investigation, Methodology, Writing – original draft. **Fozila R. Rustamova:** Formal analysis, Investigation, Methodology. **Sharadha Dayalan Naidu:** Formal analysis, Investigation, Resources. **Lauren Millichap:** Data curation, Investigation, Methodology. **Plamena R. Angelova:** Formal analysis, Investigation, Methodology, Project administration, Writing – review & editing. **Vincenzo Lariccia:** Formal analysis, Methodology, Resources, Writing – review & editing. **Albena T. Dinkova-Kostova:** Conceptualization, Formal analysis, Funding acquisition, Resources, Supervision, Writing – review & editing. **Andrey Y. Abramov:** Conceptualization, Formal analysis, Investigation, Project administration, Supervision, Writing – original draft, Writing – review & editing.

## Declaration of competing interest

The authors declare that they have no known competing financial interests or personal relationships that could have appeared to influence the work reported in this paper.

The author is an Editorial Board Member/Editor-in-Chief/Associate Editor/Guest Editor and was not involved in the editorial review or the decision to publish this article.

## Data Availability

Data will be made available on request.
